# Nectin-2 is a potential target for antibody therapy of breast and ovarian cancers

**DOI:** 10.1186/1476-4598-12-60

**Published:** 2013-06-12

**Authors:** Tsutomu Oshima, Shuji Sato, Junichi Kato, Yuki Ito, Takahiro Watanabe, Isamu Tsuji, Akira Hori, Tomofumi Kurokawa, Toshio Kokubo

**Affiliations:** 1 Pharmaceutical Research Division, Takeda Pharmaceutical Company Limited, 26-1, Muraokahigashi 2-chome, Fujisawa, Kanagawa 251-8555, Japan; 2 CMC Center, Takeda Pharmaceutical Company Limited, 4720 Takeda, Aza, Mitsui, Hikari, Yamaguchi 743-0011, Japan; 3 Present address: Center for Behavioral Molecular Genetics, University of Tsukuba, 1-1-1 Tenno-dai, Tsukuba, Ibaragi 305-8575, Japan

**Keywords:** Nectin-2, Antibody, Cancer, Antibody-dependent cellular cytotoxicity

## Abstract

**Background:**

Nectin-2 is a Ca^2+^-independent cell-cell adhesion molecule that is one of the plasma membrane components of adherens junctions. However, little has been reported about the involvement of Nectin-2 in cancer.

**Methods:**

To determine the expression of Nectin-2 in cancer tissues and cancer cell lines, we performed gene expression profile analysis, immunohistochemistry studies, and flow cytometry analysis. We also investigated the potential of this molecule as a target for antibody therapeutics to treat cancers by generating and characterizing an anti-Nectin-2 rabbit polyclonal antibody (poAb) and 256 fully human anti-Nectin-2 monoclonal antibodies (mAbs). In addition, we tested anti-Nectin-2 mAbs in several *in vivo* tumor growth inhibition models to investigate the primary mechanisms of action of the mAbs.

**Results:**

In the present study, we found that Nectin-2 was over-expressed in clinical breast and ovarian cancer tissues by using gene expression profile analysis and immunohistochemistry studies. Nectin-2 was over-expressed in various cancer cell lines as well. Furthermore, the polyclonal antibody specific to Nectin-2 suppressed the *in vitro* proliferation of OV-90 ovarian cancer cells, which express endogenous Nectin-2 on the cell surface. The anti-Nectin-2 mAbs we generated were classified into 7 epitope bins. The anti-Nectin-2 mAbs demonstrated antibody-dependent cellular cytotoxicity (ADCC) and epitope bin-dependent features such as the inhibition of Nectin-2-Nectin-2 interaction, Nectin-2-Nectin-3 interaction, and *in vitro* cancer cell proliferation. A representative anti-Nectin-2 mAb in epitope bin VII, Y-443, showed anti-tumor effects against OV-90 cells and MDA-MB-231 breast cancer cells in mouse therapeutic models, and its main mechanism of action appeared to be ADCC.

**Conclusions:**

We observed the over-expression of Nectin-2 in breast and ovarian cancers and anti-tumor activity of anti-Nectin-2 mAbs via strong ADCC. These findings suggest that Nectin-2 is a potential target for antibody therapy against breast and ovarian cancers.

## Background

Nectins are Ca^2+^-independent cell adhesion molecules that consist of 4 members: Nectin-1, Nectin-2, Nectin-3, and Nectin-4. Each member of the Nectin family except for Nectin-4 has 2 or 3 splicing variants [1]. All members except for a secreted protein Nectin-1γ have an extracellular region that contains 3 immunoglobulin (Ig)-like domains, a transmembrane region, and a cytoplasmic region. Nectins form homo-*cis*-dimers on the cell surface via their Ig2-domain [2]. The *cis*-dimers further form homo- and hetero-*trans*-dimers with other *cis*-dimers of Nectins on adjacent cells via the Ig1-domain [2-8].

Nectin-2 has been reported to regulate cell adhesion between epithelial cells through the formation of *trans*-dimers between adjacent cells [1]. Nectin-2-mediated cell adhesion induces the formation of E-cadherin-based adherens junctions followed by the formation of claudin-based tight junctions [9-12]. In addition to its function as a cell adhesion molecule, previous studies have suggested that Nectin-2 acts as an organizer of Sertoli cell-spermatid junctions in the testis [13] and of synapse formation by neurons [14,15] and as an entry receptor for viruses [16]. Nectin-2 is also known to be one of the ligands of DNAM-1 and TIGIT [17-20]. Although anti-Nectin-2 antibodies have been examined from a functional perspective, such as for the inhibition of Nectin-2 binding to DNAM-1, *in vitro* cell aggregation, or HSV-1 virion-induced cell fusion into host cells [6,18,21,22], there has been no report showing an anti-tumor effect of anti-Nectin-2 antibody. In the present study, we show that Nectin-2 is over-expressed in various cancers and that anti-Nectin-2 mAb can exert an *in vivo* anti-tumor effect on breast and ovarian cancer cells, which suggests the potential of Nectin-2 as a target for antibody therapy for cancer treatment.

## Results

### Over-expression of Nectin-2 in breast and ovarian cancers

**Figure 1:**
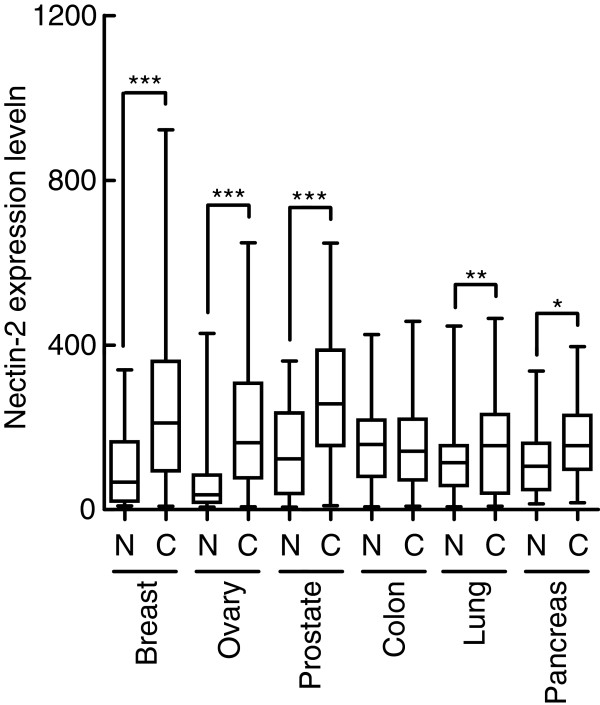
**Over-expression of Nectin-2 mRNA in cancer tissues.** A box and whisker plot of the expression level of Nectin-2 mRNA in normal tissues and cancer tissues. The vertical axis represents Nectin-2 mRNA expression level. Nectin-2 mRNA expression in the indicated tissues was analyzed using Affymetrix U_133 arrays as described in Methods. The whiskers indicate the minimum and maximum values. The box indicates the 25th–75th percentile. N, normal tissues; C, cancer tissues. ***, p < 0.0001; **, p < 0.01; *, p < 0.05 as determined by the Mann Whitney test.

**Figure 2:**
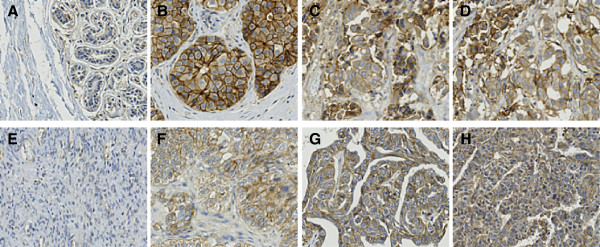
**Over-expression of Nectin-2 protein in breast and ovarian cancer tissues.** Paraffin-embedded tissue sections were stained with anti-Nectin-2 poAb as described in Methods. **A**, Normal breast tissue, **B**–**D**, breast infiltrating ductal carcinoma tissues. **E**, normal ovarian tissue, and **F**–**H**, ovarian serous carcinoma tissues.

**Figure 3:**
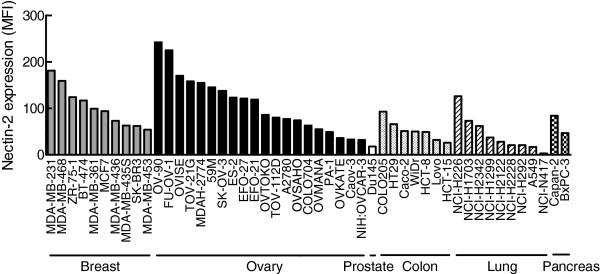
**Over-expression of Nectin-2 in cancer cell lines.** Expression levels of Nectin-2 in various human cancer cell lines were examined by flow cytometry analysis using anti-Nectin-2 rabbit poAb. The vertical axis represents Nectin-2 protein expression level indicated as an MFI of FCM analysis. MFI, median fluorescent intensity.

With the aim of identifying target proteins for antibody therapeutics, we searched for membrane-bound proteins that were over-expressed in cancer tissues compared to normal tissues using Affymetrix GeneChip Technology and found that Nectin-2 mRNA was over-expressed in various cancer tissues. An analysis that compared the mean intensity in each tissue set demonstrated that Nectin-2 was especially over-expressed in breast, ovarian, and prostate cancer tissues (Figure 1). We subsequently confirmed the over-expression of Nectin-2 protein in breast and ovarian cancer tissues by immunohistochemistry (IHC) and found that Nectin-2 protein was abundantly present in these cancer tissues while it was undetectable in normal breast and ovary tissues (Figure 2). A more extensive IHC study indicated that Nectin-2 protein was over-expressed in more than 80% of breast cancer tissue samples and approximately 50% of ovarian cancer tissues samples (Table 1). In contrast, Nectin-2 was expressed only in the liver and testis within normal tissue specimens (Table 2). In a supplemental experiment, we observed Nectin-2-expression in blood vessels (data not shown). Furthermore, Nectin-2 was broadly over-expressed in various breast and ovarian cancer cell lines when its expression was tested by flow cytometry (FCM) analysis using anti-Nectin-2 poAb (Figure 3). For the evaluation of anti-Nectin-2 antibodies in *in vitro* assays and *in vivo* studies, we selected OV-90 ovarian cancer cells, which highly expressed Nectin-2 with a median fluorescent intensity (MFI) of 242 in FCM, and MDA-MB-231 breast cancer cells (MFI = 181).

**Table 1:** Over-expression of Nectin-2 protein in breast and ovarian cancer tissues (IHC)

**Breast cancer tissue**		**Ovarian cancer tissue**	
**Type**	**Positive rate**	**Type**	**Positive rate**
Infiltrating ductal carcinoma	16/18	Serous carcinoma	22/40
Ductal carcinoma	7/7	Granular carcinoma	3/3
Infiltrating lobular carcinoma	9/9	Clear cell carcinoma	1/2
Medullary carcinoma	3/6	Endometrioid carcinoma	0/1
Mucinous adeno carcinoma	5/7	Mucinous carcinoma	1/4
Paget’s disease	7/7	Brenner tumor	1/1
	positive/total	Germinoma	0/4
		Theca cell carcinoma	1/1
		Metastasis carcinoma	1/6
			positive/total

**Table 2:** Expression of Nectin-2 protein in normal tissues (IHC)

**Tissue**	**Positive rate**	**Stained cells**
Brain	0/3	
Breast	0/5	
Heart	0/3	
Lung	0/3	
Liver	3/3	Hepatocyte
Kidney	0/3	
Spleen	0/3	
Stomach	0/3	
Colon	0/3	
Rectum	0/3	
Ovary	0/3	
Prostate	0/3	
Testis	3/3	Spermatoblast
	positive/total	

### Effect of anti-Nectin-2 poAb on OV-90 cancer cell proliferation

**Figure 4:**
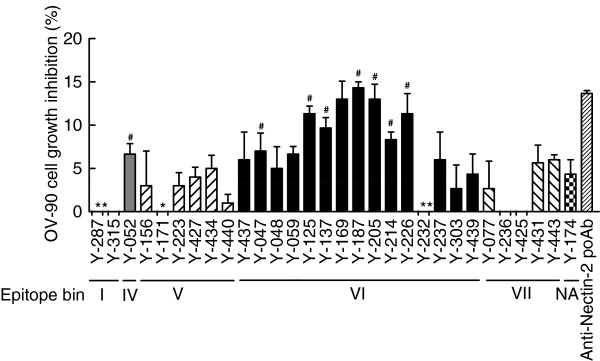
**Inhibitory activity of anti-Nectin-2 mAbs on the proliferation of OV-90 cells.** OV-90 cells were cultured in the presence of 30 μg/mL anti-Nectin-2 mAbs and poAb with 1% FBS for 6 days. The percentage inhibition of cell proliferation on the vertical axis is the mean ± S.D. of triplicate assays. *; antibodies that showed negative values for cell growth inhibition. #; antibodies that showed reproducible cell growth inhibition. NA; not assigned.

Although we found that Nectin-2 was over-expressed in various types of cancers, there has been no report suggesting the involvement of Nectin-2 in cancer cell proliferation. In order to investigate the potential of Nectin-2 as a target for antibody therapies of cancers, we generated anti-Nectin-2 rabbit poAb using recombinant Nectin-2 protein as an immunogen. The anti-Nectin-2 poAb at 30 μg/mL inhibited the proliferation of OV-90 ovarian cancer cells by 10–15% (Figure 4). Although the percentage of cancer cell growth inhibition was insignificant, the suppression was reproducible. Therefore, we decided to generate anti-Nectin-2 mAbs to further evaluate the potential of antibody therapy to treat Nectin-2 over-expressing cancers.

### Generation of fully human anti-Nectin-2 mAbs

We generated fully human anti-Nectin-2 mAbs by conventional hybridoma technology using KM mice that carried the complete locus for the human immunoglobulin heavy chain and a transgene for the human immunoglobulin kappa light chain [23]. We immunized the KM mice with a recombinant protein of the Nectin-2 extracellular domain and/or recombinant Nectin-2 over-expressing cells. As a result of the immunization and screening by a cell enzyme-linked immunosorbent assay (ELISA) using Nectin-2/CHO cells, 256 hybridoma clones that secreted human anti-Nectin-2 mAbs were established. The IgG mAbs were purified from a small-scale culture of the hybridomas and were used for characterization.

**Figure 5:**
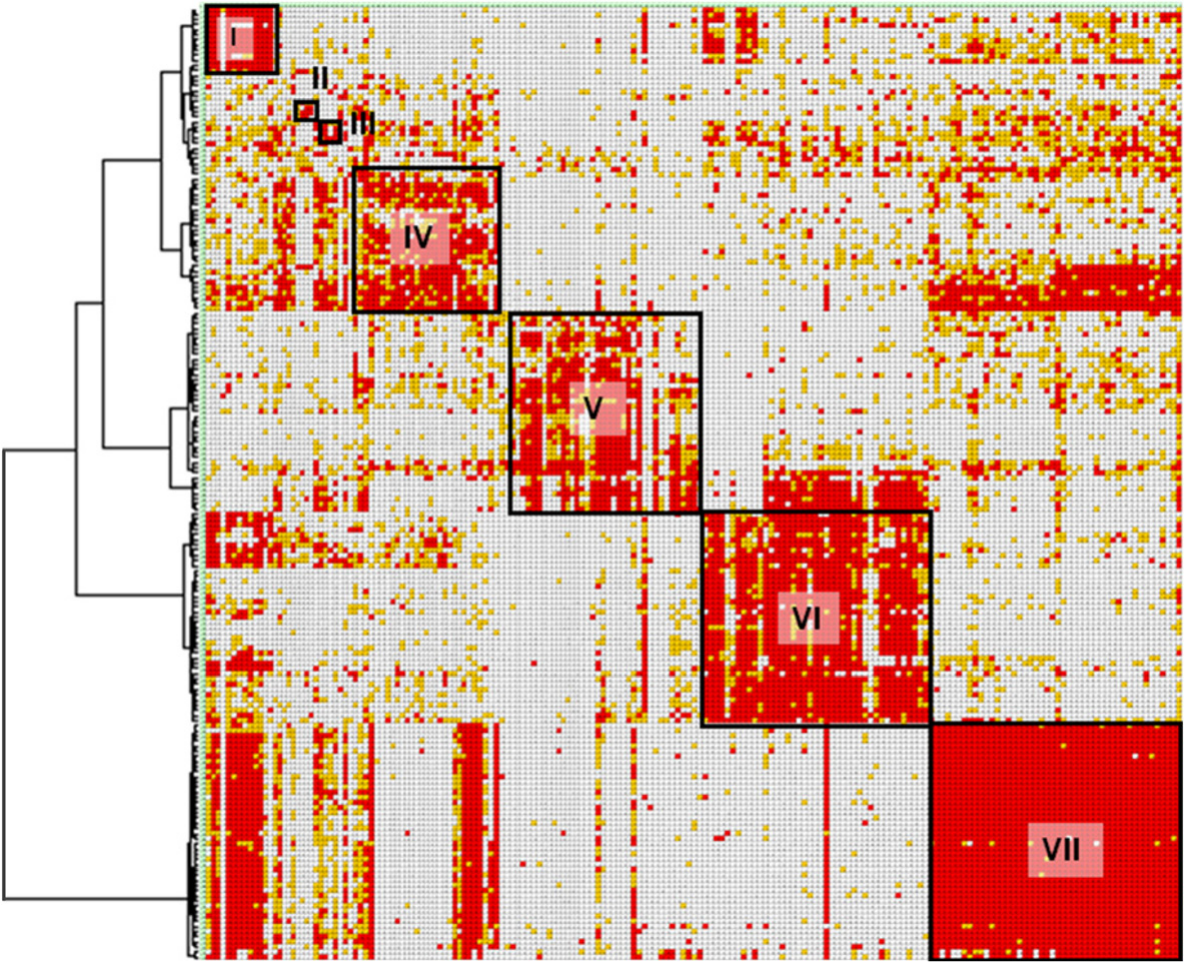
**Epitope binning of anti-Nectin-2 mAbs.** Each column and row in the matrix represents an unlabeled and biotinylated anti-Nectin-2 mAb, respectively. The red, yellow, and white cells show a combination of antibodies that showed > 70%, > 50%, and < 50% competitive binding inhibition, respectively. The dendrogram shown on the left side of the matrix was obtained by a cluster analysis using SpotFire DecisionSite for Lead Discovery.

We selected 185 out of 256 mAbs that showed relatively higher binding affinity to Nectin-2/CHO cells in the cell ELISA for an epitope binning analysis. Binding of biotinylated anti-Nectin-2 mAbs to the recombinant CHO cells that over-expressed Nectin-2 was measured in the absence and presence of an excess amount of unlabeled anti-Nectin-2 mAbs. Based on the percentage of competitive binding inhibition for each combination of antibodies, we classified the mAbs into 7 major bins (I to VII) by a clustering analysis method using Spotfire DecisionSite for Lead Discovery (Figure 5).

### Inhibitory activities of anti-Nectin-2 mAbs on the *in vitro* proliferation of OV-90 cancer cells

**Figure 6:**
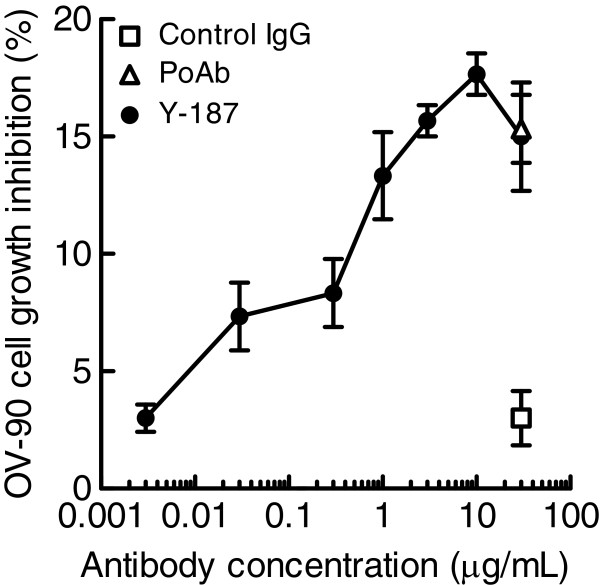
**Inhibitory activity of anti-Nectin-2 mAb Y-187 on the proliferation of OV-90 cells.** OV-90 cells were cultured in the presence of the indicated concentrations of Y-178, anti-Nectin-2 poAb, or control IgG with 1% FBS for 6 days. The percentage inhibition of cell proliferation on the vertical axis is the mean ± S.D. of triplicate assays.

As described above, anti-Nectin-2 rabbit poAb moderately suppressed the *in vitro* proliferation of OV-90 ovarian cancer cells. We next screened the 256 anti-Nectin-2 mAbs in the cell proliferation assay. The 30 mAbs that showed certain growth inhibition activities against OV-90 cells in the first screening were retested in the same assay and 8 of them reproducibly inhibited cell proliferation (Figure 4). All 8 clones (Y-052, Y-047, Y-125, Y-137, Y-187, Y-205, Y-214, and Y-226) inhibited OV-90 cell proliferation in a concentration-dependent manner. All of these mAbs except Y-052 belonged to epitope bin VI (Figure 4). The concentration-dependent inhibitory activity of the representative mAb Y-187 on OV-90 cell proliferation is shown in Figure 6 as an example.

### Inhibitory activity of anti-Nectin-2 mAbs on Nectin-2-Nectin-2 or Nectin-2-Nectin-3 interaction

**Figure 7:**
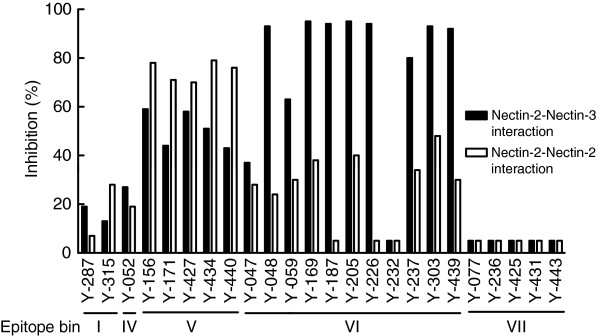
**Inhibitory activity of anti-Nectin-2 mAbs on Nectin-2-Nectin-2 and Nectin-2-Nectin-3 interaction.** Inhibitory activity of anti-Nectin-2 mAbs on Nectin-2-Nectin-2 interaction and Nectin-2-Nectin-3 interaction were measured by using an ELISA-based time-resolved fluorescence spectroscopy assay and a Biacore assay, respectively. The percentage inhibition of Nectin-2-Nectin-2 or Nectin-2-Nectin-3 interaction on the vertical axis was calculated as described in Methods.

As a cell adhesion molecule, Nectin-2 forms a *cis*-dimer and then interacts with another Nectin-2-*cis*-dimer or Nectin-3-*cis*-dimer on adjacent cells to form a homo-*trans*-dimer or hetero-*trans*-dimer, respectively [2-8]. Previous reports showed that some anti-Nectin-2 antibodies inhibited cell aggregation [6,21]. However, there has been no report that measured the effect of anti-Nectin-2 antibody separately on Nectin-2-Nectin-2 and on Nectin-2-Nectin-3 *trans*-binding. We therefore examined whether anti-Nectin-2 mAbs could inhibit Nectin-2-Nectin-2 or Nectin-2-Nectin-3 interaction using ELISA-based or Biacore-based assays, respectively. As summarized in Figure 7, most of the mAbs in epitope bins V and VI inhibited both Nectin-2-Nectin-2 and Nectin-2-Nectin-3 interactions. Interestingly, the mAb in epitope bin V inhibited Nectin-2-Nectin-2 interaction more strongly than the mAbs in epitope bin VI. In contrast, the mAbs in epitope bin VI, except Y-232, inhibited Nectin-2-Nectin-3 interaction more strongly than the mAbs in epitope bin V. The anti-Nectin-2 mAbs in epitope bins I and IV weakly inhibited interaction and the mAbs in epitope bin VII showed no inhibition of Nectin-2-Nectin-2 or Nectin-2-Nectin-3 interaction. Thus, the inhibitory activities of anti-Nectin-2 mAbs on Nectin-2-Nectin-2 and Nectin-2-Nectin-3 interactions were epitope bin-dependent.

### ADCC of anti-Nectin-2 mAbs on OV-90 cancer cells

**Figure 8:**
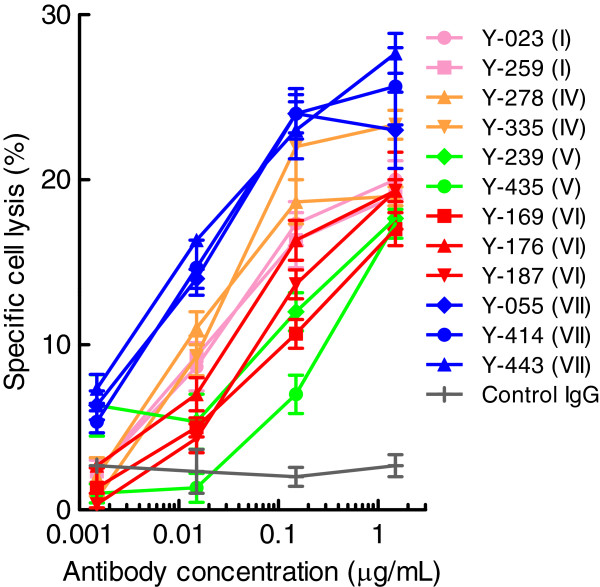
**ADCC of anti-Nectin-2 mAbs against OV-90 cells.** OV-90 cells pre-labeled with ^51^Cr were incubated with anti-Nectin-2 mAbs at a ratio of 1:50 with PBMC effector cells for 4 h at 37°C, followed by the measurement of ^51^Cr that was released into the culture supernatant. Specific cell lysis was calculated as described in Methods. The numbers in parentheses indicate the epitope bin of each antibody. The results are the mean ± S.D. of triplicate assays.

ADCC is one mechanism of action of marketed therapeutic antibodies such as rituximab and trastuzumab [24]. Among human IgG isoforms, IgG_1_ antibodies demonstrate the most potent ADCC [25]. In order to evaluate the epitope bin-dependency of anti-Nectin-2 mAbs for ADCC, we selected a few IgG_1_ antibodies with similar antigen-binding affinity (EC_50_ in cell ELISA ranging from 0.22 to 0.59 nM with the exception of Y-278 whose EC_50_ was 2.1 nM) from each epitope bin and measured the ADCC against OV-90 cells. Although the specific cell lysis was saturated at around 25%, all of the anti-Nectin-2 mAbs tested showed ADCC against OV-90 cells in a concentration-dependent manner. As shown in Figure 8, the concentrations of antibody giving 15% specific cell lysis varied roughly from 0.01 to 1 μg/mL, even though these mAbs showed similar binding affinities to Nectin-2. Interestingly, all 3 anti-Nectin-2 mAbs in epitope bin VII (Y-055, Y-414, and Y-443) appeared to show the highest potency out of all the mAbs (Figure 8).

### Anti-tumor effect of anti-Nectin-2 mAbs against OV-90 cancer cells in a mouse subcutaneous xenograft model

**Figure 9:**
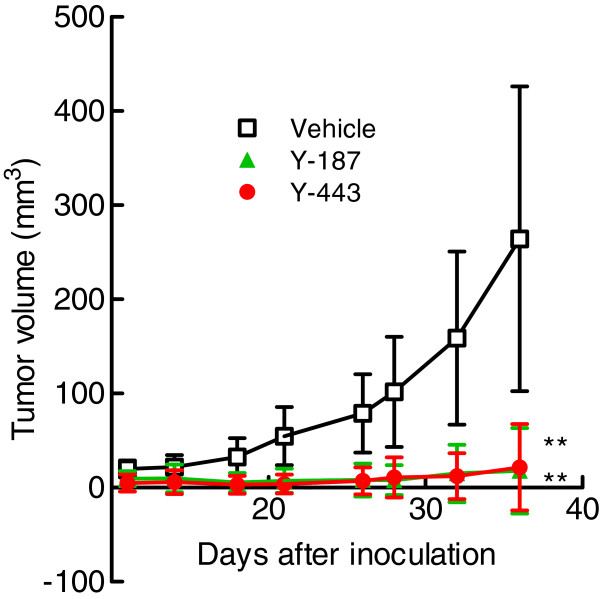
***In vivo *anti-tumor effect of anti-Nectin-2 mAbs in the OV-90 mouse subcutaneous xenograft preventive model.** OV-90 cells were subcutaneously inoculated into the flanks of nude mice. On the same day, Y-187 or Y-443 at a dose of 15 mg/kg or vehicle was intravenously administered on a biweekly basis to the mice. The results are mean ± S.D. or tumor volume. **: p < 0.001 versus the vehicle group determined by the Steel test.

To see if the anti-Nectin-2 mAbs exerted *in vivo* anti-tumor effects, we selected 2 representative mAbs, Y-187 from epitope bin VI and Y-443 from epitope bin VII, which showed different biological activities. Namely, Y-187 inhibited Nectin-2-Nectin-3 interaction and *in vitro* OV-90 cell proliferation and showed ADCC against OV-90 cells (Figures 4, 6, 7, and 8), while Y-443 did not inhibit Nectin-2-Nectin-2 or Nectin-2-Nectin-3 interaction or *in vitro* OV-90 cell proliferation, but did show ADCC (Figures 4, 7, and 8). In a subcutaneous mouse xenograft preventive model, intravenous administrations of both Y-187 and Y-443 (15 mg/kg twice a week) remarkably inhibited OV-90 tumor growth by 93% and 92%, respectively (Figure 9).

### Anti-tumor effect of Y-443 against MDA-MB-231 breast cancer cells in a mouse lung metastasis model

**Figure 10:**
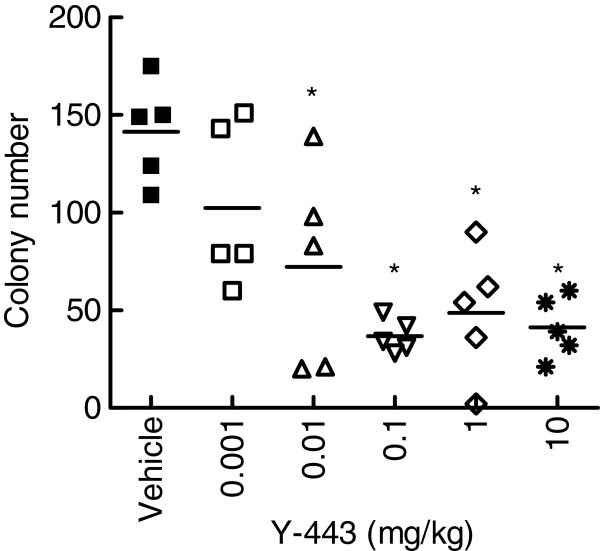
***In vivo *anti-tumor effect of Y-443 in the established MDA-MB-231 mouse lung metastasis model.** MDA-MB-231 cells were intravenously injected into nude mice. From day 33, various doses of Y-443 or vehicle were intravenously administered on a weekly basis into the mice. The results are the mean ± S.D. at day 61. *: p < 0.025, versus vehicle group as determined by the one-tailed Shirley-Williams test.

Since the inhibition of the Nectin-2 function as a cell adhesion molecule may cause adverse effects, we focused on Y-443, which did not inhibit Nectin-2-Nectin-2 or Nectin-2-Nectin-3 interaction (Figure 7), and further evaluated its anti-tumor effect on MDA-MB-231 breast cancer cells in an established mouse lung metastasis model. We started dosing the antibody at the time when the colonies of MDA-MB-231 cells became visible in the lungs. As shown in Figure 10, Y-443 suppressed the growth of MDA-MB-231 cells in lungs in a dose-dependent manner. The minimum effective dose of Y-443 for this experimental condition was 0.1 mg/kg.

### Mechanism of action of the *in vivo* anti-tumor effect of Y-443

**Figure 11:**
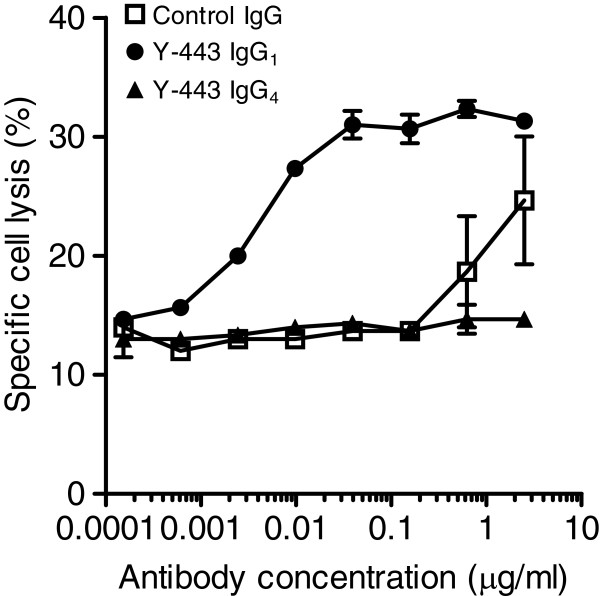
**ADCC of Y-443 and its IgG**_**4 **_**against MDA-MB-231 cells.** MDA-MB-231 cells pre-labeled with ^51^Cr were incubated with Y-443 (IgG_1_), Y-443 IgG_4_, or control human IgG at a ratio of 1:50 with PBMC effector cells for 4 h at 37°C, followed by measurement of ^51^Cr that was released into the culture supernatant. Specific cell lysis was calculated as described in Methods. The results are the mean ± S.D. of triplicate assays.

**Figure 12:**
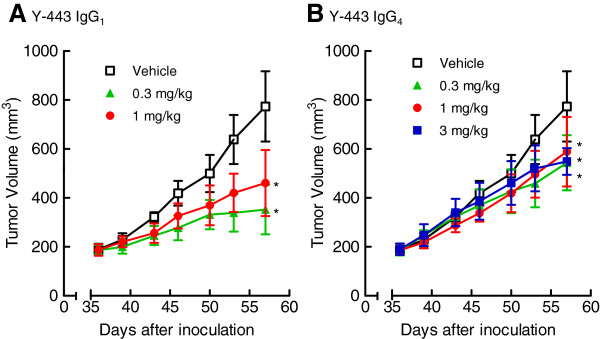
***In vivo *anti-tumor effect of Y-443 IgG**_**1 **_**versus Y-443 IgG**_**4 **_**in the established MDA-MB-231 mouse subcutaneous xenograft model.** MDA-MB-231 cells were injected into SCID mice, and the mice were then treated with Y-443 IgG_1_ (**A**) or Y-443 IgG_4_ (**B**) on days 36, 43, and 50 after cell injection (n = 5). The results are the mean ± S.D. *: p < 0.025 versus the control as determined by the one-tailed Williams test.

The differences in the *in vitro* characteristics of Y-187 and Y-443 and the similarities in anti-tumor effects on OV-90 cells described above suggested that the anti-tumor effects of these 2 mAbs were not dependent on *in vitro* inhibitory activities against cell proliferation, Nectin-2-Nectin-2 interaction or Nectin-2-Nectin-3 interaction, but were most likely dependent on ADCC. To confirm this, we prepared recombinant Y-443 IgG_4_ antibody in which the IgG_1_ Fc region of Y-443 was swapped for IgG_4_ Fc since IgG_4_ isoform is known to have much less ADCC activity [25]. The binding activity of Y-443 IgG_4_ against Nectin-2 was equivalent to that of the IgG_1_ isoform (K_D_ = 2.9 nM and 3.0 nM, respectively). Y-443 (IgG_1_) showed potent ADCC against MDA-MB-231 cells, and the ADCC disappeared as a result of Fc-conversion to IgG_4_ (Figure 11). In a mouse subcutaneous xenograft established model carrying the same breast cancer cells, the tumor growth inhibitions (TGIs) of Y-443 (IgG_1_) at 0.3 and 1 mg/kg were 65% and 53%, respectively (Figure 12). In contrast, the TGIs of Y-443 IgG_4_ at 0.3, 1, and 3 mg/kg were 39%, 31% and 39%, respectively. Thus, Y-443 IgG_4_ at 3 mg/kg showed less anti-tumor effect than Y-443 (IgG_1_) at 0.3 mg/kg.

**Figure 13:**
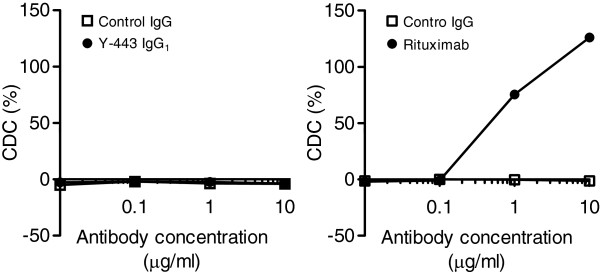
**CDC of Y-443 against MDA-MB-231 cells.** MDA-MB-231 cells and Daudi cells were incubated with Y-443 or rituximab together with human serum complement for 60 min at 37°C. The damaged cells were stained with propidium iodide. Antibody-specific CDC was calculated as described in Methods. The results are the mean ± S.D. of triplicate assays.

Tao *et al.* reported that IgG_1_ but not IgG_4_ isoform could exert complement-dependent cytotoxicity (CDC) as an effector function [26]. Therefore, we investigated the contribution of CDC in the anti-tumor effect of Y-443 (IgG_1_). However, as shown in Figure 13, Y-443 (IgG_1_) did not show CDC against MDA-MB-231 cells at all, whereas rituximab showed marked CDC against Daudi cells under similar conditions. These results suggest that the *in vivo* anti-tumor effect of the anti-Nectin-2 mAb was mainly attributed to ADCC.

## Discussion

The over-expression of Nectin-2 in breast and ovarian cancer tissues and various cancer cell lines at the mRNA and protein levels (Figures 1 and 2; Tables 1 and 2) suggests the potential of Nectin-2 as a target for antibody therapeutics to treat patients that are afflicted with these cancers. In addition, the over-expression of Nectin-2 in cell lines derived from various types of cancers (Figure 3) and in neuroblastoma, myeloid and lymphoblastic leukemias, gastric cancer, and colon cancer [27-29] suggests the possibility for the application of anti-Nectin-2 antibody to treat various cancer types.

Since a partial growth inhibition of OV-90 ovarian cancer cells had been observed using anti-Nectin-2 poAb, we generated 256 Nectin-2-specific fully human mAbs from KM mice. The mAbs were classified into 7 epitope bins by a competitive binding inhibition method using recombinant CHO cells that over-expressed Nectin-2 (Figure 5). In this assay format, 2 antibodies were classified into the same epitope bin if they bound to epitopes that were close together but were not identical and could also competitively bind to Nectin-2 due to steric hindrance. Therefore, the number of epitope bins for our anti-Nectin-2 mAbs might have been underestimated.

In the Nectin-2 interaction assay, 73 out of the 78 neutralizing mAbs belonged to epitope bin V or VI (representative data are shown in Figure 7). Interestingly, these mAbs failed to bind to a Nectin-2 Ig1 domain deletion mutant, whereas they retained the ability to bind to a Nectin-2 Ig2 domain deletion mutant (data not shown). These results demonstrate that the epitopes of antibodies in epitope bins V and VI are located in the Ig1 domain, which has been reported to mediate Nectin-2-Nectin-2 and/or Nectin-2-Nectin-3 *trans*-binding [2-8], and suggest that the antibodies in these bins may inhibit these interactions. Moreover, it is noteworthy that the antibodies in epitope bin V inhibited Nectin-2-Nectin-2 interaction more strongly than Nectin-2-Nectin-3 interaction, whereas the antibodies in epitope bin VI inhibited Nectin-2-Nectin-3 interaction more strongly than Nectin-2-Nectin-2 interaction (Figure 7). These findings suggest that the interaction sites for the hetero interaction and homo interaction of the Ig1 domain of Nectin-2 are not completely identical, which may enable Nectin-2-Nectin-2 *trans*-dimer and Nectin-2-Nectin-3 *trans*-dimer to multimerize with each other.

Anti-Nectin-2 mAbs representing each epitope bin with similar antigen binding affinity showed varying ADCC activities, and the mAbs in epitope bin VII demonstrated the strongest ADCC (Figure 8).

As mentioned previously, we found 8 anti-Nectin-2 mAbs that showed inhibitory activities in the OV-90 cell proliferation assay. Interestingly, 7 of them belonged to epitope bin VI (Figure 4) and all 7 had a similar complementarity determining region (CDR) sequence (data not shown). The results suggest that we successfully screened a subset of anti-Nectin-2 mAbs that suppressed the proliferation of OV-90 cells even though their efficacies were not marked.

The anti-Nectin-2 mAb Y-443, representing epitope bins VII, showed *in vivo* anti-tumor effects on OV-90 and MDA-MB-231 cells (Figure 9 and 10), and all the experimental results suggested that ADCC is the main mechanism of action of Y-443.

## Conclusions

This study demonstrated the over-expression of Nectin-2 in breast and ovarian cancer tissues and showed that the anti-Nectin-2 human mAb Y-443 exerts an *in vivo* anti-tumor effect on OV-90 and MDA-MB-231 cells via ADCC as the main mechanism of action. Thus, Nectin-2 was shown to be a promising target for antibody-based cancer therapies.

## Methods

### Plasmid construction

Nectin-2 was cloned from Marathon-Ready cDNA of the A549 human lung cancer cell line (BD Biosciences) into mammalian expression vectors pcDNA3.1 (Invitrogen), pEE12.4 (Lonza Biologics), and pEF1/myc-HisA (Invitrogen) to obtain pcDNA3.1-Nectin-2, pEE12.4-Nectin-2, and pEF1/myc-HisA-Nectin-2, respectively. The cDNA encoding human IgG_1_ and IgG_4_ Fc region and mouse IgG_2a_ Fc region were cloned from human spleen-derived and mouse spleen-derived Marathon-Ready cDNA (BD Biosciences), respectively. The cDNA encoding Nectin-2 extracellular domain (a.a. 1–361) fused to the human IgG_1_ Fc region and Nectin-3 extracellular domain (a.a. 1–404) fused to the mouse IgG_2a_ Fc region were inserted into pcDNA3.1 to obtain pcDNA3.1-Nectin-2-ED-hFc and pcDNA3.1-Nectin-3-ED-mFc plasmids, respectively. Similarly, the cDNA encoding C-terminal FLAG-tagged Nectin-2 extracellular domain (a.a. 1–361) and Nectin-3 extracellular domain (a.a. 1–404) were inserted into the pCMV-Tag4a plasmid (Stratagene) to obtain pCMV-Tag4-Nectin-2-ED-FLAG and pCMV-Tag4-Nectin-3-ED-FLAG plasmids, respectively. To obtain an expression plasmid encoding Y-443 with the human IgG_4_ Fc region, cDNA of the variable regions of the heavy chain or light chain of Y-443 were inserted into the GS expression vector pEE6.4 (Lonza Biologics) with human IgG_4_ Fc region cDNA or pEE12.4, respectively. The Y-443 IgG_4_ expression vector was constructed by ligation of the heavy and light chain vector.

### Cells

OV-90 and MDA-MB-231 cells were purchased from the American Type Culture Collection (ATCC). OV-90 cells were grown in a 1:1 mixture of MCDB 105 medium and Medium 199 containing 15% fetal bovine serum (FBS). MDA-MB-231 cells were grown in Leibovitz’s L-15 medium containing 10% FBS. FM3A (Health Protection Agency Culture Collections (HPACC)) cells were grown in RPMI1640 containing 10% FBS. CHO (Lonza Biologics) and NS0 cells (Lonza Biologics) were cultured in DMEM containing 10% dialyzed FBS. The other cancer cell lines for Nectin-2 expression tests for FCM analysis were purchased from the ATCC, the German Collection of Microorganisms and Cell Cultures (DSMZ), the Japanese Collection of Research Bioresources Cell Bank (JCRB), and HPACC, and were cultured according to the provider’s instructions.

To obtain Nectin-2 stable transfectants, pEF1/myc-HisA-Nectin-2 was transfected into FM3A cells by using a Gene Pulser II (Bio-Rad). The cells were cultured in 96-well plates with selection medium containing Geneticin (Life Technologies). Likewise, pEE12.4/Nectin-2 was transfected into CHO and NS0 cells using a Gene Pulser II. The transfected cells were cultured in 96-well plates with selection medium containing methionine sulfoximine and L-glutamine-free selection medium, respectively. Single colonies grown in the selection media were expanded and the expression level of Nectin-2 on the cell surface was measured by FCM using anti-Nectin-2 poAb. Thus, recombinant cell lines expressing Nectin-2 (Nectin-2/FM3A, Nectin-2/CHO, and Nectin-2/NS0) were obtained.

### Recombinant protein preparation

Recombinant proteins Nectin-2-ED-FLAG and Nectin-3-ED-FLAG were transiently expressed in FreeStyle 293 F cells (Life Technologies) by transfecting pCMV-Tag4-Nectin-2-ED-FLAG or pCMV-Tag4-Nectin-3-ED-FLAG using 293fectin (Life Technologies), respectively. The Nectin-2-ED-FLAG and Nectin-3-ED-FLAG proteins were then purified from the culture supernatant by anti-FLAG antibody column chromatography (Sigma-Aldrich) followed by a buffer exchange with Dulbecco’s phosphate-buffered saline (D-PBS) using an ultra-filtration system (Amicon). Similarly, recombinant Nectin-2-ED-hFc and Nectin-3-ED-mFc were obtained by the transfection of pcDNA3.1-Nectin-2-ED-hFc or pcDNA3.1-Nectin-3-ED-mFc, respectively. The Nectin-2-ED-hFc and Nectin-3-ED-mFc proteins were purified from the culture supernatant with Protein A Sepharose (GE Healthcare), followed by a buffer exchange with D-PBS using an ultra-filtration system.

### Anti-Nectin-2 poAb

Anti-Nectin-2 poAb was raised in New Zealand white rabbits by immunizing with recombinant Nectin-2-ED-FLAG protein emulsified with Freund’s adjuvant every 2 weeks for 3 months. The anti-Nectin-2 poAb was purified from the antiserum by affinity chromatography using a HiTrap NHS-Activated HP column (GE Healthcare) on which Nectin-2-ED-FLAG was immobilized. The purified antibodies were then passed through a Nectin-3-ED-FLAG-affinity column in order to remove antibodies that were cross-reactive to Nectin-3 or FLAG tag. The flow through fraction was used as a Nectin-2-specific poAb.

### mRNA expression analysis

Expression levels of Nectin-2 mRNA in normal tissues and cancer tissues were quantified by analyzing Affymetrix U_133 array data from Gene Logic. Nectin-2 mRNA level was calculated by multiplying each expression intensity for a given experiment (a sample hybridized onto a chip) by a global scaling factor. The scaling factor was calculated as follows: (1) from all the non-normalized expression values in the experiment, the largest 2% and smallest 2% of the values were deleted; (2) the mean of the remaining values (trimmed mean) was calculated; (3) the scale factor was calculated as 100/(trimmed mean). The value of 100 used here is the standard target value used by Gene Logic. A genechip probe 232078_at for human Nectin-2 was used for the analysis. Primary cancer tissues were used in the analysis as cancer tissues.

### IHC

The expression level of Nectin-2 protein in tissues was examined by IHC using paraffin-embedded normal tissue sections and cancer tissue sections from cancer patients (Cybrdi). The tissue sections were deparaffinized, blocked with goat serum (Vector Laboratories) for 20 min at room temperature, and incubated with 1 μg/mL anti-Nectin-2 rabbit poAb for 18 h at 4°C. After 3 washes with D-PBS, the tissue sections were incubated with ENVISION + Rabbit/HRP (Dako) for 30 min and then developed with 3, 3′-diaminobenzidine for 3 min at room temperature. The sections were counterstained with hematoxylin and mounted in Permount (Fisher Scientific). Cases with membranous staining in tumor cells were considered positive for Nectin-2 expression. The antigen-specificity of the positive samples was confirmed by staining without anti-Nectin-2 primary antibody.

### FCM

Cancer cells were incubated with 3 μg/mL anti-Nectin-2 rabbit poAb for 1 h on ice followed by incubation with 2 μg/mL Alexa488-labeled anti-rabbit IgG (Life Technologies) for 1 h on ice. Cells were washed with D-PBS containing 2% FBS after each antibody incubation step. The fluorescence intensity was measured by using a Cytomics FC 500 instrument (Beckman Coulter). The MFI ratio of the Ab-treated sample to a control sample was used as a measure of the cell surface expression level of Nectin-2 in cancer cells. A control sample was prepared by incubating cells with a rabbit IgG control (Jackson ImmunoResearch Laboratories).

### Generation of fully human anti-Nectin-2 mAbs

KM mice (10–12-weeks old, male; Kyowa Hakko Kirin) were immunized with Nectin-2-expressing cells (Nectin-2/NS0 or Nectin-2/FM3A), a recombinant protein of Nectin-2 extracellular domain (Nectin-2-ED-hFc or Nectin-2-ED-FLAG), or a combination of Nectin-2/FM3A and the recombinant protein. For cell immunization, Nectin-2/NS0 or Nectin-2/FM3A cells were pre-treated with 20 μg/mL Mitomycin C (Wako) for 30 min at 37°C, and then they were injected intraperitoneally into the mice with Ribi adjuvant at 1 × 10^7^ cells per mouse weekly for 7 weeks. For protein immunization, an emulsion of 50 μg of Nectin-2-ED-hFc or Nectin-2-ED-FLAG and Freund’s complete adjuvant (Difco) was subcutaneously injected into the KM mice. The following immunizations were repeated twice using the same amount of immunogens emulsified with Freund’s incomplete adjuvant at 2-week intervals. Two weeks after the third immunization, 10 μg of Nectin-2-ED-hFc or Nectin-2-ED-FLAG was injected into the tail vein as a final boost. For combination immunization, the first immunization was conducted subcutaneously with Nectin-2-ED-hFc or Nectin-2-ED-FLAG emulsified with Freund’s complete adjuvant followed by the second immunization with the same amount of the proteins emulsified with Freund’s incomplete adjuvant at 2-weeks intervals. In parallel, Mitomycin C treated-Nectin-2/FM3A cells were intraperitoneally injected into the mice every week for 4-weeks after the first protein immunization. Three days after the final protein immunization or 7 days after the final cell immunization, splenocytes were collected from mice that showed a high serum titer against Nectin-2 in a cell ELISA using Nectin-2/CHO and they were fused with mouse myeloma P3X63Ag8U.1 cells (ATCC) using Polyethylene Glycol 1500 (Roche Diagnostics). The fused cells were cultured in a HAT (hypoxanthine-aminopterin-thymidine)-selection media to obtain hybridomas. Hybridomas that secreted anti-Nectin-2 mAb were screened in a Nectin-2/CHO cell ELISA using the culture supernatant. After the selected hybridomas were further sub-cloned, all of the hybridomas that secreted Nectin-2-specific antibody were expanded in medium containing 10% ultra-low IgG FBS (Life Technologies) on a scale of 10–50 mL. Then, each mAb was purified from the culture supernatant using Protein A-Sepharose resin. Antibody isotypes were determined by sandwich ELISA using a plate that was separately coated with 6 different antibodies specific to human IgG_1_, IgG_2_, IgG_3_, IgG_4_, IgM or the kappa chain.

### Epitope binning study

Anti-Nectin-2 mAbs were biotinylated by using a Biotin Labeling Kit-NH2 (Dojindo). Nectin-2/CHO cells (3 × 10^3^) were pre-incubated with 5 μg/mL unlabeled anti-Nectin-2 mAb and 330 ng/mL Streptavidin-Alexa Fluor 647 (Life Technologies) in a 384-well FMAT plate (Applied Biosystems) for 10 min at room temperature. After incubation, the biotinylated anti-Nectin-2 mAb was added to the plate at a final concentration of 100 ng/mL and the plate was further incubated for 1 h at room temperature.

The fluorescence intensity of Alexa Fluor 647 on Nectin-2/CHO cells was detected by using a FMAT8200 Cellular Detection System (Applied Biosystems). The binding inhibition (%) was calculated using the following formula:

$$\mathrm{Binding}\;\mathrm{inhibition}\;\left(\%\right)=\left(1–A/B\right)\times 100$$

where A represents the total fluorescence of the added unlabeled antibody and B represents the total fluorescence in an unlabeled antibody-free well. An epitope binning study was performed with the binding inhibition data by employing Ward’s hierarchical clustering method and using SpotFire DecisionSite for Lead Discovery (TIBCO Software).

### Cell proliferation assay

OV-90 cells were seeded at 3 × 10^3^ cells/well in a 96-well plate in the presence of 1% FBS and 30 μg/mL anti-Nectin-2 poAb or mAbs and then cultured for 6 days at 37°C. Cell proliferation was measured by using Cell Counting Kit-8 (Dojindo). The efficacy of the anti-Nectin-2 mAbs was evaluated with various concentrations of the antibodies under the same conditions. Cell growth inhibition (%) was calculated using the following formula:

$$\begin{array}{ll} \mathrm{Cell}\;\mathrm{growth}\;\mathrm{inhibition}\;\left(\%\right)=100\times & [1–{\mathrm{Absorbance}}_{450}\left(\mathrm{with}\;\mathrm{Ab}\right)/\\ & {\mathrm{Absorbance}}_{450}\left(\mathrm{without}\;\mathrm{Ab}\right)[ \end{array}$$

### Nectin-2-Nectin-2 and Nectin-2-Nectin-3 interaction inhibition assays

Nectin-2-Nectin-3 interaction inhibition was quantitatively assessed using a Biacore2000 (GE Healthcare). In brief, Nectin-3-ED-mFc protein was immobilized on sensor chip CM5 (GE Healthcare) by using an Amine Coupling Kit (GE Healthcare). A mixture of Nectin-2-ED-hFc at 40 μg/mL and anti-Nectin-2 mAb at 30 μg/mL was passed through the Nectin-3-ED-mFc immobilized chip. Nectin-2-Nectin-3 interaction inhibition was calculated as a percentage of the decrease in the maximum response unit compared to a human IgG control (Jackson ImmunoResearch Laboratories).

Nectin-2-Nectin-2 interaction inhibition was measured by using an ELISA-based time-resolved fluorescence spectroscopy assay. Nectin-2-ED-hFc protein was conjugated with europium N1 ITC chelate by using a DELFIA Eu-Labeling Kit (PerkinElmer). Unlabeled Nectin-2-ED-hFc protein was immobilized on to a Delfia Clear Strip Plate (PerkinElmer). After blocking with PBS containing 2% bovine serum albumin, the Eu-labeled Nectin-2-ED-hFc and anti-Nectin-2 mAb were simultaneously added at final concentrations of 3.2 μg/mL and 30 μg/mL, respectively, followed by incubation for 1.5 h at room temperature. After washing with D-PBS containing 0.05% Tween20, Enhancement Solution (PerkinElmer) was added to each well. The fluorescence intensity was measured at 615 nm with an excitation wavelength of 340 nm and a delayed time of 400 μs by using an ARVO1420 Multilabel Counter (PerkinElmer). Nectin-2-Nectin-2 interaction inhibition was calculated as a percentage of the decrease in the fluorescence signal compared to a human IgG control.

### ADCC assay

Human peripheral blood mononuclear cells (PBMC) from AllCells were cultured in RPMI1640 medium containing 10% FBS, 0.1 nM human IL-2 (DIACLONE Research), and 55 μM 2-mercaptoethanol for 24 h. PBMC were incubated with ^51^Cr-labeled OV-90 cells or ^51^Cr-labeled MDA-MB-231 cells at a ratio of 50:1 in the presence of anti-Nectin-2 mAb for 4 h at 37°C. After incubation, the radioactivity of ^51^Cr that had been released into the culture medium from the target cells was measured by using an AccuFLEX γ 7000 (Aloka). Specific lysis by anti-Nectin-2 mAb was calculated using the following formula:

$$\mathrm{Specific}\;\mathrm{lysis}\;\left(\%\right)=100\times \left(A–B\right)/\left(C–B\right)$$

where A represents the radioactivity of the test supernatant, B represents the radioactivity of the target cells alone, and C represents the radioactivity of the maximum ^51^Cr release from the target cells that were lysed with 1% Triton X-100.

### CDC assay

MDA-MB-231 cells (5 × 10^3^) were incubated with 10-fold diluted human serum complement (Quidel) and various concentrations of Y-443 in a black wall/transparent bottom 96-well plate (BD Biosciences) for 60 min at 37°C. The cells were stained with 10 μM propidium iodide (PI), and the PI-positive cells (CDC-damaged cells) were detected using an Acumen ex3 instrument (TTP Labtech). As a positive control, rituximab (Roche) was used in combination with Daudi cells (ATCC). CDC activity was calculated using the following formula:

$$\mathrm{CDC}\;\mathrm{activity}\left(\%\right)=100\times \left(A–B\right)/\left(C–B\right)$$

where A represents the number of PI-positive cells in the presence of mAb and human serum, B represents that in the absence of mAb, and C represents the number of PI-positive cells that were incubated with 0.1% Triton X-100.

### Preparation of anti-Nectin-2 mAbs for *in vivo* study

The hybridomas that produced Y-187 and Y-443 were cultured in Daigo’s T medium (Nihon Pharmaceutical) containing 10% Ultra Low IgG FBS (Life Technologies). The culture supernatant was filtered, concentrated, and then purified with a Protein A-column. The antibody fraction was separated on a Superdex 200 26/60 column (GE Healthcare) to obtain the monomer fraction and endotoxins were removed by using an ActiClean Etox column (Sterogene). The recombinant IgG_4_ form of Y-443 was prepared as follows. CHOK1SV cells (Lonza Biologics) were maintained in CD-CHO medium (Life Technologies) containing 6 mM L-glutamine. Linearized Y-443 IgG_4_ expression vector was transfected into CHOK1SV cells with a Gene Pulser II. The cells were seeded into 96-well plates at 37°C in a humidified 8% CO2. After 1 day of culturing, methionine sulfoximine (MSX) was added at a final concentration of 25 μM. After selection of the culture, a recombinant CHOK1SV clone that produced high levels of Y-443 IgG_4_ was selected and the cells were expanded into T75 flasks in serum-free CD-CHO medium containing 25 μM MSX and then adapted into a suspension culture. Y-443 IgG_4_ was purified from the supernatant of a 1 L fed-batch culture by chromatography with Protein A Sepharose FF (GE Healthcare), followed by Superdex200 26/60.

### *In vivo* study

For the OV-90 subcutaneous mouse xenograft model, 8 × 10^6^ OV-90 cells were subcutaneously inoculated into a flank of BALB/cAJcl-nu/nu mice (6 weeks old, females) from CLEA Japan. The mice were randomly grouped (n = 13–17). Vehicle (D-PBS), Y-187, or Y-443 (15 mg/kg) was intravenously injected twice weekly for 5 weeks from the day of the cancer cell inoculation. Tumor diameters were measured with a caliper twice weekly and approximate tumor volumes were calculated as 0.5 × length × (width)^2^ during the treatment period to monitor tumor growth. The TGI was calculated by the following formula:

$$\mathrm{TGI}=100\times \left(1–A/B\right)$$

where A is the average tumor volume for the Ab-treated group and B is the average tumor volume for the vehicle group.

The difference in the average tumor volume between the Ab-treated groups and the vehicle group was analyzed using the Steel test.

For the MDA-MB-231 subcutaneous xenograft model, 3 × 10^6^ MDA-MB-231 cells were subcutaneously inoculated with Matrigel (Becton Dickinson) into BALB/cAJcl-nu/nu mice (6 weeks old, female) as described above. The mice were grouped 36 days after the inoculation when the average sizes of the tumors reached approximately 200 mm^3^ and then vehicle (D-PBS), Y-443 (0.3 and 1 mg/kg), or its IgG_4_ form antibody (0.3, 1, and 3 mg/kg) was intravenously injected once per week for 3 weeks. The tumor volumes were measured and the TGI for each treatment group was determined at day 57 as described above. The difference in the average tumor volume between the Ab-treated groups and the vehicle group was analyzed using Williams test.

The MDA-MB-231 lung metastasis model was carried out as follows. Briefly, 100 μL of 1 × 10^6^ cultured MDA-MB-231 cancer cell suspension in Hank’s buffered salt solution was intravenously injected into the tail vein of SCID mice (5–6 weeks old). The mice were randomly grouped (n = 5) 33 days after the inoculation and were treated with vehicle (D-PBS) or Y-443 (0.001, 0.01, 0.1, 1, or 10 mg/kg) weekly on days 33, 40, 47, and 54. On day 61, the mice were sacrificed to excise the lungs, and 0.2% Evans blue dye was injected intratracheally to visualize their tumor colonies. The lungs were subsequently fixed with a mixture of picric acid, 10% neutral buffered formalin, and acetic acid (15:5:1), and the number of colonies on the diaphragmatic surface of the lungs was counted macroscopically in a blind manner. The difference between the colony numbers of the Ab-treated group and the vehicle group was analyzed using the one-tailed Shirley-Williams test.

All of the animal studies including immunization were carried out in strict accordance with the recommendations in the Guide for the Care and Use of Laboratory Animals of the National Institutes of Health. The protocol was approved by the Committee on the Ethics of Animal Experiments of Takeda Pharmaceutical Company Ltd. (Permit Numbers: 2802, 2701, and E1-0311).

## Abbreviations

ADCC: Antibody-dependent cellular cytotoxicity; CDC: Complement-dependent cytotoxicity; D-PBS: Dulbecco’s phosphate- buffered saline; ELISA: Enzyme-linked immunosorbent assay; FBS: Fetal bovine serum; FCM: Flow cytometry; Ig: Immunoglobulin; IHC: Immunohistochemistry; mAb: Monoclonal antibody; MFI: Median fluorescent intensity; PBMC: Peripheral blood mononuclear cells; poAb: Polyclonal antibody; SD: Standard deviation; TGI: Tumor growth inhibition.

## Competing interests

The authors hereby declare that they have no competing interests.

## Authors’ contributions

TO, SS, and TKu designed the studies and wrote the manuscript. TO generated anti-Nectin-2 mAbs and executed Nectin-2-Nectin-2 and Nectin-2-Nectin-3 interaction assays and the cancer cell proliferation assay. SS assessed the mRNA and protein expression levels of Nectin-2, and performed the cancer cell proliferation and CDC assay. JK and YI tested the *in vivo* anti-tumor effects of the anti-Nectin-2 mAbs. TW performed the ADCC assay. IT carried out the epitope binning study. AH and TKo coordinated the studies. All of the authors have read and approved the final version of the manuscript.
